# Nomogram for Risk Prediction of Mortality for Patients with Critical Cardiovascular Disease Treated by Continuous Renal Replacement Therapy in Coronary Care Unit

**DOI:** 10.31083/j.rcm2306189

**Published:** 2022-05-26

**Authors:** Xiaoming Zhu, Kuibao Li, Mulei Chen

**Affiliations:** ^1^Heart Center and Beijing Key Laboratory of Hypertension, Beijing Chaoyang Hospital, Capital Medical University, 100020 Beijing, China

**Keywords:** continuous renal replacement therapy, survival prediction, risk of mortality, critical cardiovascular disease, nomogram

## Abstract

**Aims:**

To establish a nomogram-scoring model for evaluating the risk of death in patients with critical cardiovascular disease after continuous renal replacement therapy (CRRT) in a coronary care unit (CCU).

**Methods:**

This retrospective cohort study included data collected on 172 patients, in whom CRRT was initiated in the CCU between January 2017 and June 2021. Predictors of mortality were selected using an adaptive least absolute shrinkage and selection operator logistic model and used to construct a nomogram. The nomogram was evaluated using the concordance index (C-index) and Hosmer–Lemeshow test.

**Results:**

The number of patients who died in-hospital after CRRT was 91 (52.9%). The results of the multivariate logistic regression analyses clarified that age, history of hypertension and/or coronary artery bypass grafting, a diagnosis of unstable angina pectoris or acute myocardial infarction, ejection fraction, systolic blood pressure, creatinine, neutrophil, and platelet counts before CRRT initiation were significant predictors of early mortality in patients treated with CRRT. The nomogram constructed on these predictors demonstrated significant discriminative power with an unadjusted C-index of 0.902 (95% CI: 0.858–0.945) and a bootstrap-corrected C-index of 0.875. Visual inspection showed a good agreement between actual and predicted probabilities (Hosmer–Lemeshow χ^2^ = 5.032, *p*-value = 0.754).

**Conclusions:**

Our nomogram based on nine readily available predictors is a reliable and convenient tool for identifying critical patients undergoing CRRT at risk of mortality in the CCU.

## 1. Introduction

Continuous renal replacement therapy (CRRT) is the most commonly used form of renal replacement therapy for the treatment of critically ill patients with acute kidney injury (AKI) or end-stage kidney disease (ESKD) with hemodynamic instability, significant electrolyte or acid–base, disbalances or volume overload [1, 2]. Although only 5–10% of all patients, irrespective of whether the patients have AKI or ESKD, are treated with CRRT because of great fluid volume control and hemodynamic stability in the intensive care unit (ICU) [3, 4], mortality in such patients is high (50–60%) [5, 6]. Simultaneously, CRRT also increases the pain experienced by patients, the risk of infection or hemorrhage with the requirement of anticoagulation, medical costs, and the household economic burden [7]. Some additional critical factors should be considered while deciding whether to initiate CRRT, including the patient’s demographic data, socioeconomic status, and clinical circumstances [8]. Thus, developing a comprehensive assessment tool to predict the risk of mortality for each patient is necessary.

In particular, most patients in the cardiac care unit (CCU) are affected with cardiac insufficiency, which directly affects renal perfusion, and water and sodium retention will increase the cardiac capacity load. However, because patient characteristics and the number of prognostic factors present in individual patients may vary and the prognostic factors may mutually interact, accurately evaluating the outcome of patients undergoing CRRT in the current clinical scenario is difficult. As a useful means to elucidate patient characteristics, outcomes, prognostic factors, and comprehensive risk, simple scoring models that include the prognostic factors identified by multivariable analysis have been developed for several diseases. Applying these models has led to a better understanding and management of AKI in the past [9, 10, 11, 12, 13], whereas no specialized tool is currently available for patients with severe heart disease undergoing CRRT because of the characteristic of unstable circulation.

Based on the aforementioned statement, our study aimed to analyze the conventional data in the medical system that could be used to identify significant predictors of mortality, validate a cost-efficient nomogram for developing a standardized assessment tool to determine the prognosis, and improve the clinical management of patients with the critical cardiovascular disease treated with CRRT.

## 2. Materials and Methods 

### 2.1 Patient Enrollment 

The data of 172 patients treated with bedside continuous veno venous hemofiltration (CVVH) in the CCU of Beijing Chaoyang Hospital between January 1, 2017, and June 30, 2021, were collected retrospectively. The inclusion criteria of this retrospective cohort study were as follows: (1) adults (aged ≥18 years) and (2) treated with CVVH in the CCU. If the same patient has multiple hemofiltrations during hospitalization, only the data of the first time will be recorded. Three patients treated with CRRT in January 2017 before the electronic medical record system of our hospital was upgraded were excluded to avoid unclear and nonstandard records caused by handwriting that could affect the accuracy of the data (**Supplementary Fig. 1**). This study was approved by the Ethics Committee of Beijing Chaoyang Hospital.

### 2.2 Data Collection 

Data of patients’ demographics, history, physical examination, laboratory examination results, medication history, and lifestyle were collected upon admission to the hospital. Medical histories comprising type-2 diabetes, hypertension, coronary disease, cerebrovascular disease, chronic kidney disease (CKD), and heart failure were reviewed and extracted. Laboratory data including complete blood counts, serum biochemical tests for the kidney, and heart, procalcitonin, and coagulation dysfunction, were obtained from the laboratory records. Echocardiography was performed to record cardiac functional parameters, including ejection fraction (EF), within the first 24 hours after admission. We also recorded the blood test results and blood pressure before conducting hemofiltration. Furthermore, Acute Physiology and Chronic Health Evaluation II (APACHE-II) scores and outcomes of disease (in-hospital mortality) were recorded.

### 2.3 Statistical Analysis 

As appropriate, continuous data are expressed as mean ± SD or median (interquartile range), based on the normality of data distribution tested by the Kolmogorov–Smirnov test. Comparisons between groups were performed using independent-sample *t*-tests or Mann–Whitney U tests, as appropriate. Categorical data are presented as counts and percentages and were compared using the Chi-square test or Fisher’s exact test, as appropriate. The adaptive least absolute shrinkage and selection operator (LASSO) logistic model was used to identify critical determinants of all-cause mortality, and the optimal value of λ was determined via 10-fold cross-validations. The performance of the models was evaluated based on discrimination and calibration.

Model discrimination was expressed as the area under the receiver operating characteristic curve (AUC). The internal consistency of the discrimination performance measures was evaluated by the bootstrapping method. Calibration was assessed using the Hosmer–Lemeshow goodness-of-fit test and visualized with calibration plots. Decision curve analysis (DCA) was used to assess the clinical usefulness of the model. Based on the logistic regression model, a reduced multivariate model was used to create a nomogram, presenting a specific system for calculating the risk of mortality. Non-parametric missing value imputation, based on the MissForest procedure in R, was applied to impute missing data. A random forest model based on the remaining variables in the dataset was constructed to predict the missing values with an estimation of the internally cross-validated errors. The *p*-values were 2-sided and an α level of 0.05 was considered statistically significant. All analyses were conducted using R version 4.0.2 (https://www.r-project.org/).

## 3. Results 

### 3.1 Patient Characteristics 

A total of 172 patients (121 men, 52 women; median age: 69.5 [interquartile range (IQR): 58–78 years] who met the inclusion criteria were stratified by their complications. The full description of the patients at baseline is presented in Table 1. Among them, 81 patients (47.1%) were discharged from the hospital upon recovery, and 91 (52.9%) died. We identified 32 risk factors for in-hospital mortality among critically ill patients-treated with CRRT. In the entire cohort, 58% had baseline (CKD) and 46 patients (27%) received routine hemodialysis. Hypertension (83%), acute coronary syndrome (ACS; 73%), and heart failure (31%) were the leading comorbidities in our patients; 16.5% of those patients who died (15/91) had a history of hemofiltration prior to hospitalization, and 38.3% (31/81) of those who were alive at the end of hospitalization had undergone prior hemofiltration (*p* = 0.002). Percutaneous coronary intervention history showed an almost significant difference between survivors (26%) and non-survivors (13%) (*p* = 0.054). The two cohorts had similar laboratory test results, except the counts of white blood cells, neutrophils, platelets, platelet division width and creatinine results assessed on admission compared with the pre-CRRT data.

**Table 1.** **Demographic and clinicopathological characteristics in the patients**.

Characteristic		Total	Survivors	Non-survivors	*p*-value
		(n = 172)	(n = 81)	(n = 91)	
Sex, n (%)					
	Male	121 (70)	56 (69)	65 (71)	0.861
	Female	51 (30)	25 (31)	26 (29)	
Age (IQR)		69.5 (58, 78)	65 (53, 72)	74 (64.5, 81.5)	<0.001
History					
Acute coronary syndrome, n (%)					0.001
	No	47 (27)	32 (40)	15 (16)	
	Yes	125 (73)	49 (60)	76 (84)	
Heart failure, n (%)					0.254
	No	111 (65)	49 (60)	62 (68)	
	Yes	54 (31)	30 (37)	24 (26)	
	Missing	7 (4)	2 (2)	5 (5)	
Chronic kidney disease, n (%)					0.012
	No	70 (41)	25 (31)	45 (49)	
	Yes	100 (58)	54 (67)	46 (51)	
	Missing	2 (1)	2 (2)	0 (0)	
Hypertension, n (%)					0.198
	No	29 (17)	10 (12)	19 (21)	
	Yes	143 (83)	71 (88)	72 (79)	
Diabetes, n (%)					0.378
	No	90 (52)	39 (48)	51 (56)	
	Yes	82 (48)	42 (52)	40 (44)	
Old myocardial infarction, n (%)					0.277
	No	137 (80)	62 (77)	75 (82)	
	Yes	33 (19)	17 (21)	16 (18)	
	Suspected	2 (1)	2 (2)	0 (0)	
Stroke, n (%)					0.725
	No	136 (79)	62 (77)	74 (81)	
	Cerebral infarction	34 (20)	18 (22)	16 (18)	
	Cerebral hemorrhage	2 (1)	1 (1)	1 (1)	
Percutaneous coronary intervention, n (%)					0.054
	No	139 (81)	60 (74)	79 (87)	
	Yes	33 (19)	21 (26)	12 (13)	
Coronary artery bypass surgery, n (%)					0.309
	No	163 (95)	75 (93)	88 (97)	
	Yes	9 (5)	6 (7)	3 (3)	
Atrial fibrillation, n (%)					0.652
	No	154 (90)	73 (90)	81 (89)	
	Paroxysmal	9 (5)	3 (4)	6 (7)	
	AF				
	Sustained AF	9 (5)	5 (6)	4 (4)	
Routine hemodiafiltration, n (%)					0.002
	No	126 (73)	50 (62)	76 (84)	
	Yes	46 (27)	31 (38)	15 (16)	
Out-of-hospital cardiac arrest, n (%)					0.324
	No	147 (85)	72 (89)	75 (82)	
	Yes	25 (15)	9 (11)	16 (18)	
Results from the first evaluation on admission					
Creatinine (μmol/L) (IQR)		304.8 (138, 557.9)	426.65 (158.35, 604.5)	233.8 (112.3, 497.7)	0.022
White Blood Cell (${\mathrm{\times 10}}^{9}$/L) (IQR)		8.8 (6.86, 13.06)	7.69 (6.04, 10.94)	9.79 (7.38, 13.91)	0.01
Neutrophilic Granulocyte Percentage (%) (IQR)		81.8 (70.75, 87.75)	80.1 (70.8, 86.7)	83.35 (70.58, 88.68)	0.297
Lymphocyte Count (%) (IQR)		11.4 (6.12, 18.08)	12 (6.9, 17.4)	10.9 (5.5, 18.7)	0.59
Hemoglobin (g/L)		107.34 ± 28.68	104.14 ± 29.92	110.22 ± 27.36	0.169
Platelets (${\mathrm{\times 10}}^{9}$/L) (IQR)		175 (134.5, 230.5)	168 (124, 217)	179.5 (138.25, 234.25)	0.253
Platelet Distribution Width (fl) (IQR)		11.9 (10.62, 13.67)	11.5 (10.38, 12.5)	12.15 (11.03, 14.2)	0.03
Mean Platelet Volume (fl) (IQR)		10.6 (9.9, 11.5)	10.35 (9.8, 11.2)	10.7 (10.03, 11.6)	0.061
Ejection Fraction (%) (IQR)		45 (37.75, 58)	52 (39, 60)	43 (35, 55.5)	0.012
Results from the day before CRRT initiation					
Heart Rate (beats/min) (IQR)		87.5 (74, 100)	85 (72.5, 95)	88 (75, 102.5)	0.145
Systolic Blood Pressure (mmHg)		120.06 ± 22.16	127.89 ± 21.82	113.27 ± 20.22	<0.001
Creatinine (μmol/L) (IQR)		444.9 (293.18, 643.03)	533 (332.48, 666.22)	413.15 (246.75, 548.68)	0.002
White Blood Cell (${\mathrm{\times 10}}^{9}$/L) (IQR)		11.38 (7.73, 15.86)	9.85 (6.29, 13.26)	12.5 (9.79, 16.41)	<0.001
Neutrophilic Granulocyte Percentage (%) (IQR)		86.9 (79.45, 90.4)	82.8 (72.6, 88.6)	88.3 (83.8, 91.1)	<0.001
Lymphocyte Count (%) (IQR)		6.85 (4.4, 11.48)	9 (5.45, 15.9)	5.6 (4.1, 9.5)	0.001
Hemoglobin (g/L) (IQR)		89.5 (75, 108)	90 (77, 105.5)	89 (75, 110)	0.957
Platelets (${\mathrm{\times 10}}^{9}$/L) (IQR)		153 (101.75, 212.5)	155 (102, 217.5)	151 (101, 210)	0.361
Platelet Distribution Width (fl) (IQR)		12.7 (11.2, 14.35)	12.15 (10.72, 13.78)	13.4 (11.9, 14.7)	0.002
Mean Platelet Volume (fl) (IQR)		11.1 (10.3, 11.8)	10.85 (10, 11.57)	11.3 (10.7, 11.8)	0.013
APACHE II (IQR)		20 (14, 28)	14 (11, 18)	28 (23.25, 30)	<0.001

All categorical values are expressed as n (%). All continuous values are expressed as mean ± SD. IQR, inter-quartile range.

### 3.2 Variable Selection for Constructing a Prediction Model and a Nomogram 

**Fig. 1.**
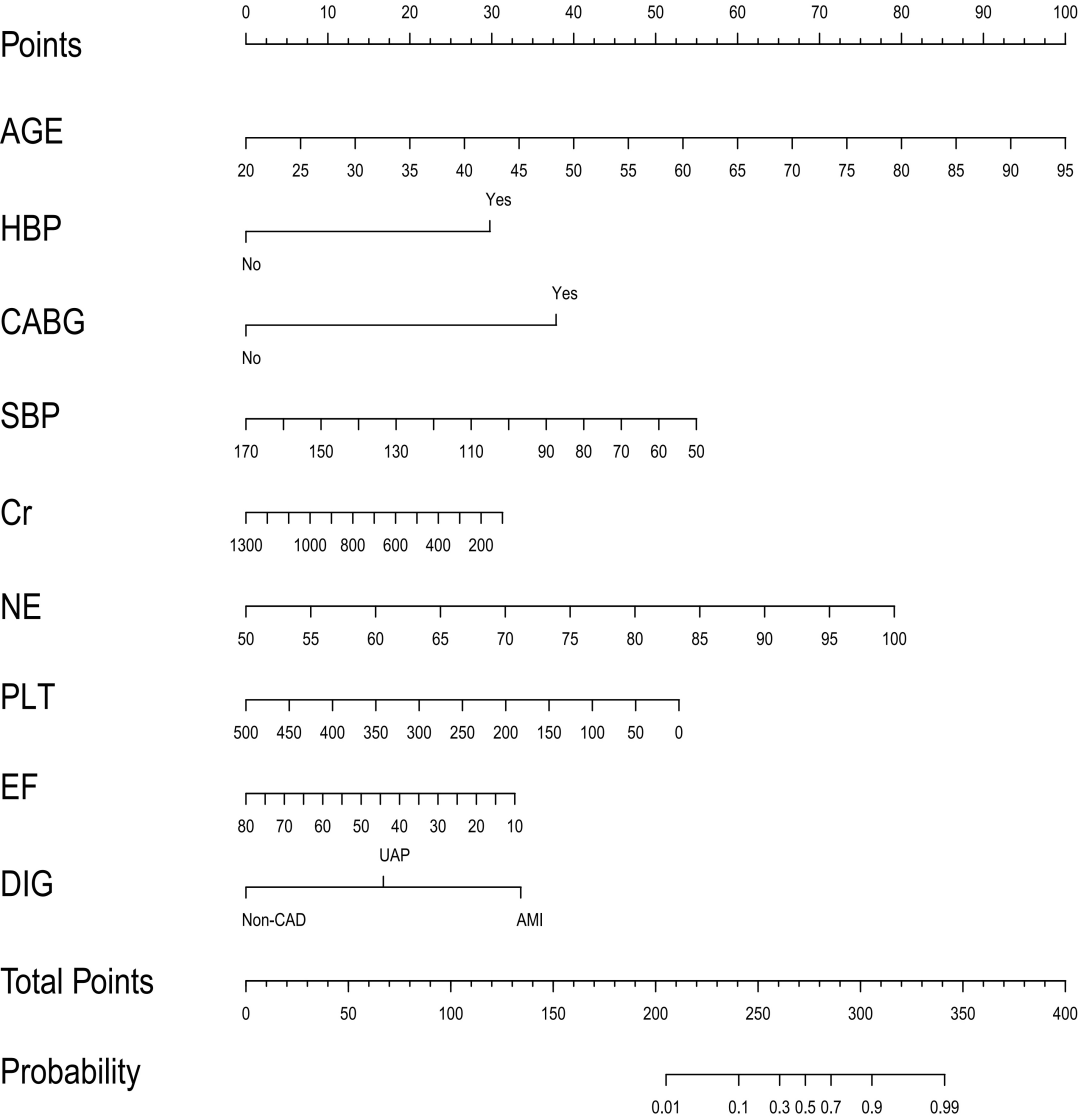
**The nomogram for predicting in-hospital mortality of patients undergoing CRRT**. HBP, hypertension history; CABG, coronary artery bypass grafting history; SBP, systolic blood pressure before CRRT initiation; Cr (creatinine), NE (neutrophils), PLT (platelets): laboratory tests results before CRRT initiation; EF, ejection fraction; DIG, diagnosis (non-coronary heart disease, Unstable Angina Pectoris or Acute Myocardial Infarction).

The results suggested the presence of multiple modifiable factors increased the probability of in-hospital mortality in critical patients treated with CRRT. For LASSO regression models, we chose the most regularized and parsimonious model with a tuning λ (log scale), giving a cross-validated error within one standard error of the minimum, which reduced 34 variables in Table 1 to 9 factors that were associated with in-hospital all-cause mortality of patients undergoing CRRT. Those 9 critical factors were age, hypertension, a diagnosis of unstable angina pectoris, non-ST-elevation myocardial infarction or ST-elevation myocardial infarction, history of coronary artery bypass grafting, EF, systolic blood pressure (SBP), and baseline clinical markers including creatinine level, neutrophil count, and platelet counts before CRRT initiation. By integrating these 9 relevant factors of mortality, we constructed a predictive nomogram for predicting the in-hospital mortality of patients undergoing CRRT (Fig. 1).

### 3.3 Model/Nomogram Performance

**Fig. 2.**
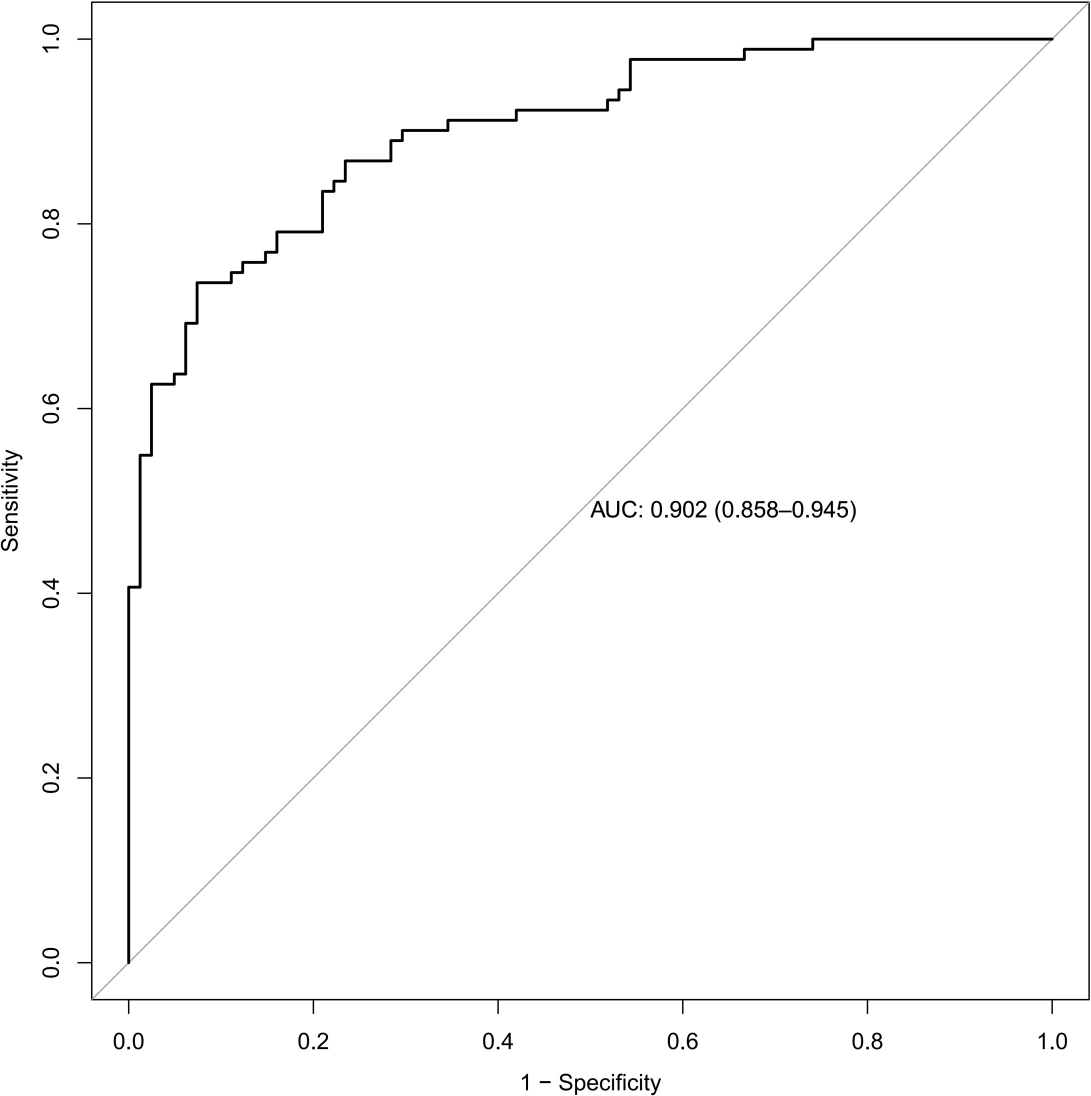
**Receiver operating characteristic curve of the model for predicting the risk of mortality showed that our model had excellent discrimination**. Area under ROC curve (AUC): 0.902 (95% CI: 0.858–0.945).

To assess the discrimination of our model, a receiver operating characteristic curve was plotted, which showed that our model had excellent discrimination [AUC: 0.902 (95% CI: 0.858–0.945)] (Fig. 2). After bootstrap validation (200 repetitions), the corrected C-index (i.e., corrected AUC) was 0.875. The calibration curve (**Supplementary Fig. 2**) shows that our model also had a good agreement between actual and predicted probabilities, and this agreement was reinforced by the Hosmer–Lemeshow test results (χ^2^ = 5.032, *p*-value for lack-of-fit = 0.754). The DCA graphically showed that our risk model provided a larger net benefit across the range of mortality risk (**Supplementary Fig. 3**).

## 4. Discussion

In the present study, we developed a simple and easily utilizable nomogram for predicting the risk of in-hospital mortality for patients undergoing CRRT in a cohort of CCU-admitted patients. Based on the demographic, clinical, and laboratory data extracted from the electronic medical records, our visualized nomogram with nine variables (age, history of hypertension and/or CABG, diagnosis of unstable angina pectoris or myocardial infarction, EF, SBP, and clinical markers, including creatinine, neutrophil, and platelet counts before initiating CRRT), demonstrated a good degree of differentiation that facilitates the early identification of patients at high risk of mortality, in whom timely intervention would be necessary.

The mortality rate was particularly high within hours or days following CRRT initiation, mainly due to the deterioration of the patients’ condition when CRRT is started. This study demonstrated that in-hospital mortality was high among critically ill patients treated with CRRT (52.9%). Therefore, it is necessary to find a way to predict individuals with a high mortality risk, who could have devastating outcomes following CRRT. Once the individuals with a high risk of non-survival are identified, physicians and patients can understand the prognosis better. The need for timely adjustment of the treatment plan should also be reconsidered to avoid unnecessary harm to the patients and reinvent the wheel.

Several risk prediction models for the incidence of mortality after CRRT have been developed for various populations. An existing meta-analysis demonstrated that older age, lower body mass index, higher APACHE-II and sequential organ failure assessment (SOFA) scores, lower SBP and diastolic blood pressure, decreased serum creatinine levels, and increased serum sodium levels were significantly associated with increased in-hospital mortality in critical cases undergoing CRRT [14]. However, two studies confirmed and extended this association between CRRT-associated mortality and some risk factors [7, 15]. Another study externally validated previous models. Most models were found to be poorly calibrated, and the SOFA score outperformed the APACHE-II score in predicting the outcomes of patients with AKI in a critical condition undergoing CRRT [16]. The common flaw in these studies is that they only identified isolated risk factors and did not integrate the results to quantify the risk to determine whether the risk of death was high or low.

Considering the heterogeneity of the population, we developed a nomogram for patients with critical cardiovascular diseases, which is provided to combine the predict factors directly. By using multivariable logistic regression analysis, it seems that elderly patients and those with coronary disease, regardless of a previous CABG surgery [17] or newly diagnosed ACS, have an increased mortality risk following CRRT. Clinically, it was found that cardiac and renal function were significantly lower in patients with ACS than in the population without cardiovascular diseases. Patients with chronic renal insufficiency exhibit a pronounced risk for cardiovascular events [18], while myocardial infarction and heart failure with reduced EF can lead to AKI due to pump exhaustion and renal hypoperfusion. The severity of AKI is determined by the elevation of serum creatinine levels, which, globally, is a key determinant to start renal replacement therapy [19]. On the other hand, our study showed that low levels of serum creatinine at CRRT initiation increased the risk of in-hospital mortality, which is consistent with previous studies [14]. This implies that patients with normal or mildly elevated creatinine may have serious volume overload caused by heart failure and require CRRT treatment. It is well known that as an indication for hemofiltration, volume overload is mostly caused by renal insufficiency and sodium retention due to acute kidney injury or acute left heart failure. Its severity affects patient outcomes but is difficult to quantify. In the data entry stage, we tried to use the 24-hours input and output volume, and Killip grade to represent the degree, but they did not become predictive factors eventually. The reason may be that volume overload is a common clinical sign in patients with hemofiltration. Once the hemofiltration initiates volume load is going to be improved and relieved.

Hypertension is another risk factor that affects kidney function. Owing to the disordered cardiac microenvironment, the renin-angiotensin-aldosterone system is overactive, leading to increased cardiac output with high cardiac load, reduced renal vascular blood flow, and aggravated renal ischemia [20]. Systolic blood pressure, a hemodynamic parameter, was significantly lower in patients who exhibited early mortality compared with survivors following CRRT initiation. Our study also revealed that decreased SBP was associated with mortality. In the CCU, CRRT treatment is primarily considered for patients in critical condition, who are hemodynamically unstable, have cardiovascular instability, and/or suffer from volume overload due to acute left heart failure. In addition to paying close attention to the control of pressure and volume loads, thrombocytopenia induced by sepsis or intra-aortic balloon pump, and combined therapy with anticoagulant, antiplatelet and thrombolytic drugs also increase the risk of hemorrhage which result in increased mortality [21]. In our study, non-survivors had lower platelet counts at the time of CRRT initiation, which is consistent with previous studies. Simultaneously, patients with thrombocytopenia are at a higher risk of bleeding during CRRT, which needs to be further assessed by the nomogram prudently. Another important factor in the nomogram is neutrophils, innate immune phagocytes, which play a role in the elimination of pathogens, immune regulation, immunity, and infection prevention, especially in sepsis and acute myocardial infarction in the CCU. Sepsis is a common cause of hospitalization and death in the ICU [22, 23], which can induce multiple organ dysfunction and even failure [24].

Our results also showed that a high neutrophil count could be associated with an increase in mortality and negatively impact on the prognosis [25]. This may be because the CRRT process can eliminate inflammatory mediators in the body, regulate the immune system, and improve renal function, with good clinical efficacy [26, 27]. Hence, the inflammatory response in the body is reduced and the metabolic circulation and excretion of the body are increased. The improvement of the inflammatory state in patients with early CRRT before infection can protect renal tubules from further injury, which is conducive to the recovery of organ function and lowers mortality. A previous study suggested that higher neutrophil counts were associated with a decreased filter life [28]. Correspondingly, CRRT with advanced sepsis was of little prognostic significance. In conclusion, our nomogram only includes nine predictors and shows a good discriminative power, with AUCs >0.8 in the development cohort and was internally validated; the nomogram is convenient and can be useful for clinical reference.

A previous study has validated a nomogram for predicting in-hospital mortality in patients undergoing CRRT with five predictors including patient age, days after admission, lactic acid level, blood glucose concentration, and diastolic blood pressure [29]. Among these factors, blood glucose concentration is greatly under the influence of various factors, especially closely related to eating, so it cannot be regarded as a reliable factor. Complications such as blood pressure decrease, tachycardia, and other arrhythmias can occur during CRRT and worsen the clinical situation of patients suffering from severe heart disease [30]. Through the analysis of patients in the particular situation-CCU department, we did not find that days after admission is a predictor. By contrast, based on the above analysis of the nine factors, our nomogram is clinically applicable and simple to use, with a good discriminative power that facilitates the early identification of patients at high risk of mortality. Nevertheless, some limitations were present in our study. First, we cannot rule out selection bias because of this is a single-center study with relatively small sample size. For this reason, the selection and evaluation of predictors may be affected. The results still need to be validated, but as a preliminary attempt, it may help to predict the risk of death before CRRT is used. Therefore, a multicenter study with a larger sample size and prolonged observation time is warranted in the future to verify the factors associated with mortality incritical patients treated with CRRT. Second, other potential factors associated with early mortality in such patients, as indications for CRRT initiation, time spent on CRRT, or time since admission to the hospital, are worth further study. Rapid deterioration of cardiac function, arrhythmias and electrolyte disorder can make patients’ condition turn to a precipitous decline, so the initiation time of CRRT is very important. A timely and decisive beginning of CRRT can improve the prognosis of patients, and once the best time is missed, irreversible damage will be caused to patients. Analysis of available data suggests that hemofiltration duration cannot be an independent predictor. Subsequently, we will expand the sample size and specifically conduct data analysis for time point selection to improve the evaluation system. Third, a causal relationship could not be established as the study was observational and only the associations between several clinical factors and early or very early mortality in patients undergoing CRRT were investigated. Lastly, although our model showed a good C-index in the internal validation, no external validation was performed in this study, and no other group of critical patients without CRRT treatment was included as a control group, which would be necessary for the future to confirm our findings.

## 5. Conclusions

Our nomogram with nine predictors is a simple and viable tool for mortality risk stratification among patients with the severe cardiovascular disease treated by CRRT, which enables clinicians to identify at-risk individuals and adopt timely specific preventative measures. The prognosis of patients can be effectively understood using a nomogram. However, further external validation is required before clinical generalization.

## Data Availability

Data are available on reasonable request.

## Supplementary Material

Supplementary material associated with this article can be found, in the online version, at https://doi.org/10.31083/j.rcm2306189.
