# Supplementary material for: Nomogram for Risk Prediction of Mortality for Patients with Critical Cardiovascular Disease Treated by Continuous Renal Replacement Therapy in Coronary Care Unit
Source: Rev Cardiovasc Med. 2022 May 26;23(6):189. doi: 10.31083/j.rcm2306189 (PMC11273656; doi:10.31083/j.rcm2306189)
Supplement: Supplementary file 1 [file 2153-8174-23-6-189-s1.zip › 2153-8174-23-6-189-s1.docx]

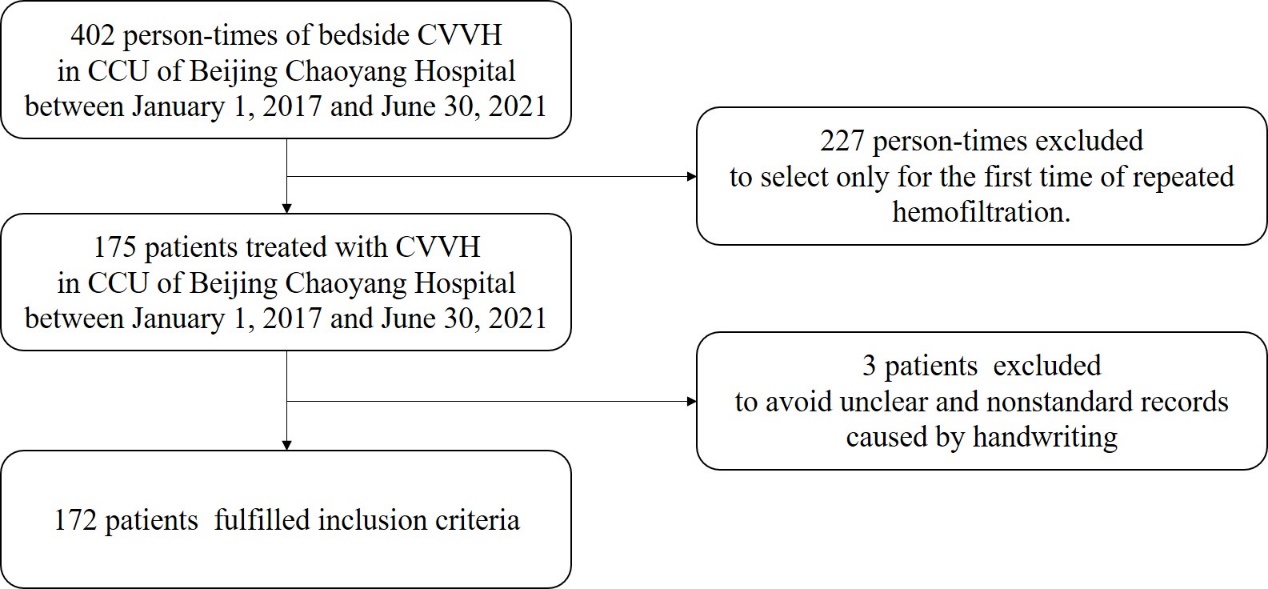


Supplementary Fig. 1. The flow chart of screened patients.

**
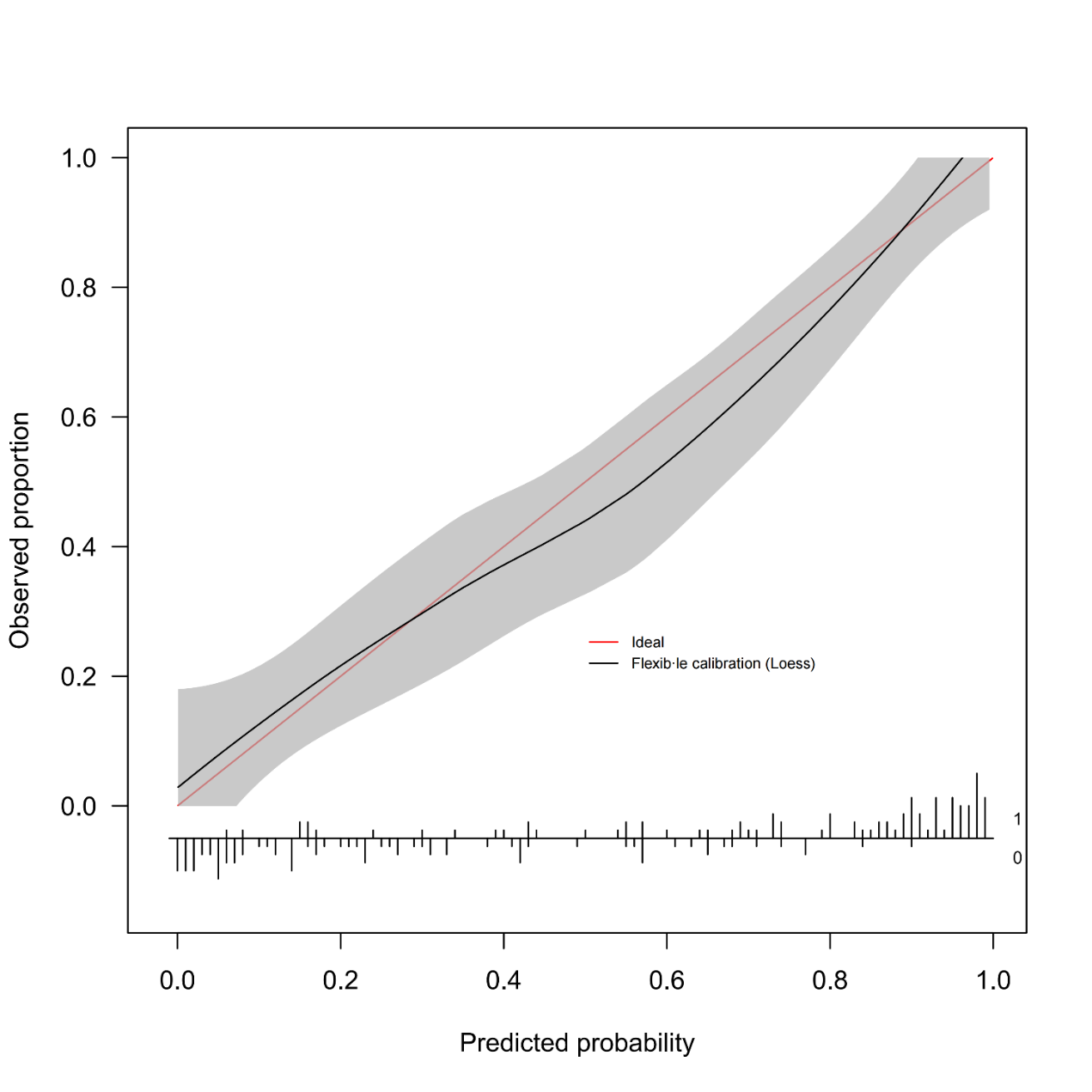
**

Supplementary Fig. 2. The calibration curve. Hosmer–Lemeshow test (χ2 = 5.032, P value for lack of fit = 0.754).


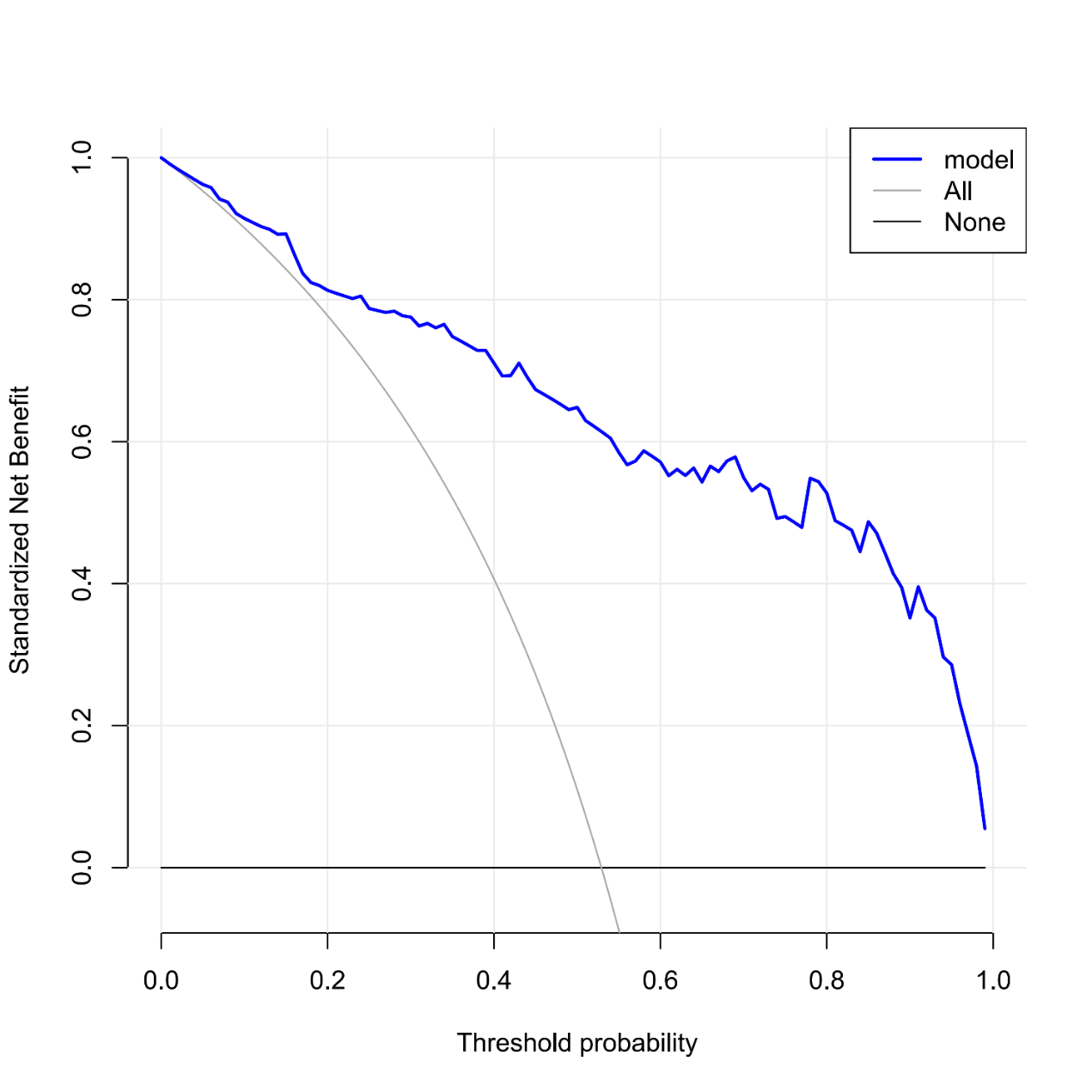


Supplementary Fig. 3. Decision curve for model.
